# In Memory of Dr. Samuel Wardle

**Published:** 1894-02

**Authors:** 


### In Memory of Dr. Samuel Wardle.

Dr. Samuel Wardle died January 24th, at his home in Hartwell, a suburb of Cincinnati, aged seventy-two years. For two months the Doctor had been a great sufferer with catarrhal pneumonia.

Dr. Wardle was born January 2, 1822, at Leicestershire, England, came to America at the age of nine years, and settled with his parents in Philadelphia, where he was educated. At the age of about twenty-two he began the study of dentistry at Philadelphia, under Dr. Elisha Neall, who was at that day one of the prominent dentists of Philadelphia. Under this teacher, Dr. Wardle had superior facilities for gaining a knowledge of the art and practice of his profession. After his graduation in 1845, he established an office in that city, where he continued to practice for four years, when he removed, coming to Cincinnati. Upon his arrival here he established a Dental Depot, on Walnut Street, near Sixth. After continuing in this for a year or two and finding it less congenial to his taste, he here established an office, and entered upon the practice of his profession which he followed successfully to within two or three months of his death.

Dr. Wardle, though well equipped in all the particulars of his profession, was especially so in the prosthetic line, and hence he gave a large share of his professional attention to supplying artificial substitutes for the natural teeth and the surrounding parts. In this he had but few if any equals, as an evidence of which it is sufficient to mention the fact that many dentists throughout the West have been for many years dependent upon him for their finest specimens of artificial dentures.

In 1847 Dr. Wardle married Margaret Anna Little, of Philadelphia, who, with four children, survives him. These are Charles W. Wardle, D.D.S., of Maysville, Ky., Samuel H. Wardle, D.D.S., Harry, and Miss Lillie Wardle remain at home.

Dr. Wardle has rendered public service in a variety of ways, and always with honor to himself. He was for a time in the army during the Civil War of this Country. There his service was so well approved that he was soon promoted to the position of 1st Lieutenant of his company. He has also been for many years a member of the Odd Fellows and Masonic Fraternities, taking a very active and prominent part in the work of these orders, and was at various times promoted to high positions because of his efficient service and his deservedly high popularity. He was a member of the Methodist Church, and well did he sustain the character of a noble, christian gentleman, esteemed and beloved by all who knew him.

His family will have the sincere sympathy of all his friends, neighbors, and acquaintances.

